# Prevalence and attributes of criminality in patients with schizophrenia

**DOI:** 10.5249/jivr.v7i1.635

**Published:** 2015-01

**Authors:** Abolfazl Ghoreishi, Soleiman Kabootvand, Ebrahim Zangani, Shahrzad Bazargan-Hejazi, Alireza Ahmadi, Habibolah Khazaie

**Affiliations:** ^a^Department of Psychiatry, Social Determinant of Health Research Center, Zanjan University of Medical Sciences, Zanjan, Iran.; ^*b*^Zanjan University of Medical Sciences, Zanjan, Iran.; ^*c*^Department of Psychology, College of Medicine at Charles Drew University of Medicine and Science & David Geffen School of Medicine at UCL A Los Angeles, CA, USA.; ^*d*^Department of Anesthesiology, Imam Reza Hospital, Kermanshah University of Medical Sciences, Kermanshah, Iran.; ^*e*^Department of Public Health Sciences, Division of Social Medicine, Karolinska Institute, Stockholm, Sweden.; ^*f*^Sleep Disorders Research Center, Kermanshah University of Medical Sciences, Kermanshah, Iran.

**Keywords:** Prevalence, Criminality, Schizophrenia

## Abstract

**Background:**

Existing research in law and psychiatry point to schizophrenia as a risk factor for violence and offense behaviors. The present study aims to: 1) report on the prevalence and types of offensive or criminal acts in patients with schizophrenia; 2) identify attributes of schizophrenic offenders; and 3) examine factors associated with offensive or criminal behaviors within a sample of schizophrenic offenders.

**Methods:**

This was a cross-sectional study of 358 patients with schizophrenia who were admitted to a psychiatric ward in Iran between 2004 and 2008. Study data was collected using patients’ medical, criminal records, as well as via personal interview with the family member. Study variables included criminality or offensive behavior, types of schizophrenia (paranoid vs. nonparanoid), experiencing hallucination, disease onset, and patients’ demographics.

**Results:**

Of the sample, 64.8% were male, 80.7% were 45 years old or younger, and 74.1% were either single or divorced. Slightly over 59 % were offenders with criminal status, of which, 9.8% were legal offenders and 48.6% were hidden offenders. The results of unadjusted logistic regression between these variables and criminality show, except for employment, marital status, and opium use, all other variables were statically associated with criminality.

**Conclusions:**

Methodological difficulties arising from this study, as well as, the role of mental health professionals, family, and legal system for prevention of violence in and by patients with schizophrenia are discussed.

## Introduction

Schizophrenia is a form of chronic psychosis that may include bizarre behavior, paranoia, anxiety, delusions, withdrawal, and suicidal tendencies.^1^ The causes of schizophrenia are unknown, but there is evidence of heredity factors and imbalance in brain chemistry. Its prevalence between men and women is equal, and two million new cases of this disease appear around the world each year. ^2^

A crime is an act that is prohibited by law through penalty. According to Goldoozian, for human behavior to be considered a crime, three elements are necessary: 1) Legally, the criminal act should be prohibited by law; 2) materially, the criminal act should be executed or realized; and 3) “spiritually,” the criminal act should be accompanied by criminal intention or guilt. These three elements must be present for an act to be labeled as a crime.^3^

The association between schizophrenia and committing violent acts or different forms of crime is evident in literature, encompassing interpersonal attack and murder.^4-7^ Compared to their healthy counterparts in the general population, individuals diagnosed with schizophrenia are 4 to 6 times more likely to commit a violent crime.^8^ In Western countries, 6% of the homicide perpetrators in the populations were labeled schizophrenic.^9^ Existing research also indicates that the prevalence of crime in patients with schizophrenia is significantly associated with male sex, being single, refusing to accept treatment, substance abuse and duration of illness. ^10-12^

The present study brings two different branches of science together, namely law and psychiatry, to: 1) report on the prevalence and types of offensive/criminal acts in patients with schizophrenia; 2) identify attributes of schizophrenic offenders; and 3) examine factors associated with criminal behaviors within a sample of schizophrenic offenders. 

## Methods

**Study Design and Data Collection**

This was a cross-sectional study of 358 patients with schizophrenia who were admitted to a psychiatric ward in Zanjan, Iran between 2004 and 2008. Zanjan is the capital of Zanjan Province in northwestern Iran. In 2011 its population was estimated at 386, 851 and it is considered the 20th largest city in Iran. Study data were collected using patients’ medical and criminal records, as well as via personal interview with the family members. 

**Study Variables**

To measure criminality or offensive behavior, we borrowed the categories used by the Iranian legal system: offender, hidden offender, and having no criminal record.^3^ Based on these categories, patients were grouped into criminal (including both legal and hidden offenders) vs. non-criminals. Addiction data were extracted from the medical chart indicating whether a patient has addiction to alcohol or narcotics. Measurement variables for mental illnesses were the types of schizophrenia (paranoid vs. nonparanoid), experiencing hallucination (yes vs. no), and disease onset (including early vs. late onset). Other measures included demographics (e.g., age, gender, level of education, employment status, and marital status). The Zanjan University of Medical Sciences Institutional Review Board approved all procedures.

**Data Analysis**

Univariate analysis was used to report pattern of criminology in the sample and illustrate the overall characteristics of the sample with respect to the study variables. Unadjusted logistic regressions were performed to identify predictors of criminality at bivariate level. Data were analyzed using IBM SPSS Statistics 20. Odds ratios and 95% confidence intervals (CIs) are presented and statistical significance was set at P values ≤ 0.05.

## Results

Of the 358 patients, 59.2% (n=149) were offenders with criminal status. Of these, 9.8% (35) were legal offenders and 48.6% (174) were hidden offenders (data not shown). Table 1 presents the overall characteristics of the sample. With respect to demographic characteristics of the sample, 64.8% were male, 80.7% were 45 years old or younger, 74.1% were either single or divorced, and 71.5% had less than a high school education. Alcohol was abused by 57.5% of the sample, and 95.0% had a record of using opium on a daily basis. With respect to mental health, 68.2% were experiencing hallucinations. 63.4% were suffering from paranoid type vs. 36.6% who suffered from nonparanoid type (Table 1). Table 2 illustrates a breakdown of the types of offenses patients were engaged in during their illness period. Of 10 offensive behaviors ranging from premeditated homicide to setting fire, spouse abuse (41.9%), damage to property (26.8%), and child abuse (24.0%) were the top three offensive behaviors highly prevalent in this sample. 

**Table 1:** Sample overall characteristics (N = 358).

Variables	F(%)
**Gender**	
-Male	232(64.8)
-Female	126(35.2)
**Crime**	
-Non offender	146(40.8)
-Offender	212(59.2)
**Patient characteristics (%)**	
**Age**	
16-25	52(14.5)
26-35	143(39.9)
36-45	94(26.3)
46-55	43(12.0)
56-65	21(5.9)
66-75	5(1.4)
**Marital status**	
-Married	93(26.0)
-Single	186(52.0)
-Divorced	79(22.1)
**Employment**	
-Employed before schizophrenia	153(42.7)
-Unemployed after schizophrenia	46(12.8)
-Unemployed before schizophrenia	159(44.4)
**Education**	
-Illiterate	83(23.2)
-Secondary school	173(48.3)
-High school graduate	42(11.7)
-Pre-university	48(13.4)
-University	12(3.4)
**Alcohol abuse**	
-Yes	206(57.5)
-No	152(42.5)
**Opium abuse**	
-Yes	340(95.0)
-No	18(5.0)
**Hallucination**	
-Yes	244(68.2)
-No	114(31.8)
**Type of Schizophrenia**	
-Paranoia	227(63.4)
-Nonparanoid	131(36.6)
**Disease onset**	
-Early onset	180(50.3)
-Late onset	178(49.7)

**Table 2:** Distribution patterns of different types of offenses in the sample.

Type of Offense	F (%)
Premeditated Homicide	3(0.8)
Children abuse	17(4.7)
Child abuse	86(24.0)
Spouse abuse	150(41.9)
Damage to property	96(26.8)
Insult	30(8.4)
Assault	35(9.8)
Theft	13(3.6)
Rape	1(0.3)
Setting fire	1(0.3)

Table 3 presents the results of unadjusted logistic regressions to identify factors that are associated with having a criminal status. Column 3 in the tables indicates that male vs. female (64.7% vs. 49.2%), younger vs. older patient (71.6% vs. 7.2%), more educated patients vs. less educated (68.0% vs. 37.3%), those employed before diagnosis of schizophrenia vs. unemployed (98.0% vs. 39.0%), married vs. single (100.0% vs. 55.9%) and divorced (19.0%) were more engaged in criminal behaviors. Similarly a higher percentage of offenders were alcohol abusers (72.8%), opium users (62.4%), experienced hallucinations (66.4%), were paranoid type (66.1%), and experienced early onset of schizophrenia (83.3%). The unadjusted association between these variables and criminality is present in the last two columns of Table 3. Except for employment, marital status, and opium use, all other variables were statically associated with criminality (P <0.05). The results of unadjusted logistic regressions between these variables and criminality are presented in the last two columns of Table 3. Except for employment, marital status, and opium use, all other variables were statically associated with criminality (P < 0.05). 

**Table 3:** Unadjusted logistic models predicting committing crime versus not committing in the sample (n=358).

Independent Variables	No Crime Non-offenders	Committed Crime Offenders	Unadjusted OR (95% CI)	P-value
**Demographics**				
**Gender**				.005
-Male	82(35.3)	150(64.7)	1.88 (1.21-2.93)	
-Female	64(50.8)	62(49.2)	Reference:	
**Age**				.000
-16-45	82(28.4)	207(71.6)	32.3 (12.5-32.1)	
-46-75	64(92.8)	5(7.2)	Reference	
**Education**				.000
-Illiterate/some high school	82(32.0)	38(37.3)	3.57 (2.21-5.77)	
-High school or more	64(62.7)	174(68.0)	Reference:	
**Employment**				.000
-Employed before schizophrenia	3(2.0)	150(98.0)	0.13(.004-.042)	
-Unemployed before schizophrenia	97(61.0)	62(39.0)	Reference:	
**Marital status**				.186
-Married	0(0.00)	93(100.0)	---	
-Single	82(44.1)	104(55.9)	1.33(.871-2.03)	
-Divorced	64(81.0)	15(19.0)	Reference	
**Addiction Characteristics and Mental Illness**				
**Alcohol Abuse**				.000
-Yes	56(27.2)	150(72.8)	3.88 (2.89-6.07)	
-No	90(59.2)	62(40.8)	Reference:	
**Opium**				---
Yes	128(37.6)	212(62.4)		
No	18 (100.0)	0(0.0)	---	
**Illusion**				.000
-Yes	82(33.6)	162(66.4)	2.52 (1.60-3.98)	
-No	64(56.1)	50(23.9)	Reference	
**Type of schizophrenia**				.001
-Paranoid	77(33.9)	150(66.1)	2.16(1.39-3.36)	
-Nonparanoid	69(52.7)	62(47.3)	Reference	
**Disease onset**				.000
-Early onset	30(16.7)	150(83.3)	0.107 (0.01-0.17)	
-Late onset	116(65.2)	62(34.8)	Reference:	

## Discussion

Our findings point to a high prevalence of criminality among the patients with schizophrenia in the study. In addition, the overwhelming majority of the offenders were engaged in hidden offenses facing no legal consequences. A closer look at the data shows that 97% of victims of the hidden-offenders were family members. This is alarming since people often have a difficult time reporting offensive behaviors committed by family members with mental disorders, fearing stigmatization or persecution by the legal system. The complexity of this issue should remind health professionals to be more proactive in understanding the interplay of schizophrenia and offensive behaviors in the family setting and suggest violence prevention strategies and provide adequate referrals. 

The existing evidence supports our findings.^13-15^ A review study using national and international data concluded an increased risk of criminality in schizophrenia patients ^16^ and suggested a greater need to integrate an understanding of aggressive behaviors and schizophrenia in present-day psychiatry. Others have reported that inadequate attention to association between criminality and schizophrenia consequentially generates substantial increase in admissions of such patients to forensic mental hospitals, ^17-18^ and inappropriate patient shifting to the judicial system. ^19^

Similar to our findings, other studies have attributed an increased risk of violent offense in schizophrenia to younger age, male sex,^20^ co-morbid substance abuse, ^21-24^ experiencing hallucination, ^25^ and early onset of the disease. 

National and international researchers have found that male sex predicates earlier onset, different symptomology, and poorer disease course and outcome of schizophrenia, as well as violent behaviors due to the interplay of sex hormones and neurodevelopmental and psychosocial sex differences.^26-30^

The findings of substance abuse studies suggest that the risk of criminality and violent behaviors in comorbid patients with schizophrenia with substance abuse is similar to that of substance abusing individuals without schizophrenia, suggesting that violence reduction efforts should consider focusing on primary and secondary substance abuse prevention strategies.^10,31^ Also, since crime is most often the offender’s response to delusions or hallucinations ^25^ the significance of early treatment of delusions and hallucinations should be underscored. ^32^

Age at onset of psychosis may carry clinical significance across psychotic disorders and appears to be associated with specific genetic abnormalities; specifically, differential clinical and behavioral features, and notably, a history of lifetime cannabis abuse/dependence and violence.^33,34^ Clinicians are in a unique position to screen for and identify risks associated with the manifestation of violence between early and late-onset schizophrenia. ^34^

Methodological difficulties arising from this study limit the interpretation of the findings on the association between offensive behavior/criminality and schizophrenia. This includes a lack of clear data on the diagnostic tools used in the sample, use of a different definition of illegal behavior in Iran, and use of data from one psychiatric site. In addition, a small sample size limited our ability to perform subgroup analyses and use rigorous tests to identify the independent predictors of criminality or offensive behaviors. These limitations restrict the generalizability of our findings to other populations of patients with schizophrenia. Nevertheless, our findings add to the body of knowledge regarding this topic, which is rarely investigated in low- and middle-income countries.^35^ And like most national and international studies, this study shows a high percentage of criminal activities among patients with schizophrenia and an association, albeit statistically limited, between criminality and some measures of socio-demographics, substance abuse, and type of schizophrenia. 

Larger and longer longitudinal studies are needed to provide better knowledge of the association between the stage of schizophrenia and type of offense committed by such patients in the context of the patient’s biological, psychosocial make up, as well as institutionalization and deinstitutionalization experiences. Findings from such studies could support appropriate and timely actions regarding the prevention, treatment and management of this disease and its association with criminality. Early assessment and identification of violent behaviors in patients with schizophrenic-spectrum disorder by qualified psychiatrists who are familiar with the national legal or professional guidelines on this issue can be cost and time saving. It also may assist the risk assessment process in schizophrenia. It could also prevent premature passing of the patient to the jail and psych wards, which could be more traumatic and damaging to a vulnerable person.^36^

## Conclusion

The present study was an attempt to delineate relevant statistics concerning schizophrenia and criminality. Five years of data on patients with schizophrenia show a high percentage of offensive/criminal behaviors in these patients. More specifically, hidden offenses and those that target family members were prevalent. These findings, although limited, point to the potential association between criminality and gender, age, education, substance abuse, type of schizophrenia, and disease onset among schizophrenia patients. 

While more replication of this study is warranted to substantiate our results, they are informative for early detection and outreach services especially to family and criminal justice settings. In middle-income countries such as Iran, adequate community-based facilities and outreach services can result in lifting the burden of schizophrenia-related violence in families and patients, and reduce the financial cost of premature hospitalization. 

Mental health professionals can play a crucial role in raising awareness of and communicating with family members the risk of violent behaviors in patients with schizophrenia. They can also raise community awareness regarding the potential victimization of patients with schizophrenia.^20,37^ They can promote the notion that people with major mental health disorders need highly empathetic and supportive therapies to keep them safe. People with schizophrenia who are actively paranoid or experiencing hallucinations may be vulnerable, and living within a system or society that labels anything unknown or different as bad/wrong could only aggravate their treatment. 

Further interventional studies are needed to report on the criminality outcome of integrated and comprehensive care for patients with schizophrenia living in the community. There are also limited local studies on the attitudes of mental health professionals and their inclination to integrate aggressive behavior into their understanding of psychosis.^17^ Furthermore, a lack of knowledge on behalf of the judicial system regarding the mental state of the accused may cause an increasing numbers of patients with schizophrenia-spectrum disorders to be referred to forensic psychiatric institutions, raising concerns regarding efforts to destigmatize both patients and psychiatry. 
